# Targeting effector proteins of plant pathogens as a strategy for durable plant disease resistance

**DOI:** 10.3389/fmicb.2025.1681047

**Published:** 2025-11-14

**Authors:** Govindasamy Senthilraja, Maddi Sandhya, Eswaran Priyadharshini, Theerthagiri Anand, Murugavel Kavitha, Nagendran Tharmalingam

**Affiliations:** 1 Department of Plant Pathology, Centre for Plant Protection Studies, Tamil Nadu Agricultural University, Coimbatore, Tamil Nadu, India; 2 Horticultural Research Station, Tamil Nadu Agricultural University, Udagamandalam, Tamil Nadu, India; 3 Department of Medicine, Houston Methodist Research Institute, Houston, TX, United States

**Keywords:** effectors, S genes, CRiSPR/Cas, RNAi, decoys, plant immunity, durable resistance, food security

## 1 Introduction

Microbes (fungi, bacteria, and viruses) are the major cause of plant diseases and are responsible for devastating yield reductions that translate into enormous economic burdens. Global annual losses with regard to plant diseases account for $220 billion (Savary et al., 2019), posing a significant threat to global food security (Sharma et al., 2020). Various strategies have been used to address these losses. For example, traditional breeding approaches help to provide crops with durable resistance, yet it is constrained by the rapid breakdown of resistance and the limited availability of resistant genes (R genes) in the host plant. However, pathogens can overcome that resistance over time. Additionally, chemical pesticides may be used, but most pathogens gain resistance through repeated and often widespread application (Meade et al., 2021). At the molecular level, pathogens, including bacteria, fungi, and viruses, produce effector molecules, which are proteinaceous biological molecules that act as mediators of interaction with the host plant. Effector molecules are released into the apoplast or host cell, thereby helping the pathogen subvert the host's immune response (Liu et al., 2014). These molecules are critical virulence determinants, found mainly in the secretion system of bacteria, haustoria of fungi, and salivary secretions of insects that transmit diseases caused by viruses and phytoplasmas (Gonzalez et al., 2016).

Biotechnology tools have been leveraged to target effectors for plant disease management. These approaches offer specificity and provide long-term resistance to the host (Belete and Boyraz, 2019). In this paper, we highlight the potential of effector binding sites as molecular targets that can be leveraged using techniques such as CRISPR/Cas-based genome editing, RNA interference, decoy engineering, and effectoromics approaches. These approaches involve identifying genes that will accelerate resistance breeding and ultimately contributing to sustainable disease management and food security.

## 2 Discussion

### 2.1 Effectors as key components in disease development

Plant pathogenic effectors play a crucial role in the interaction between host and pathogens. These specialized molecules facilitate pathogen colonization and nutrient extraction by modulating host cellular processes (Harris et al., 2023). They modify levels of various phytohormones to promote pathogenicity and evade plant immunity (Han and Kahmann, 2019). Effectors are classified as intracellular or extracellular based on their site of localization. Intracellular effectors are released into the cytoplasm or nucleus, where they suppress plant immunity. Extracellular effectors operate outside the cell, in the apoplast, breaching the physical and chemical barriers of plant defense (De Wit, 2016). Translocated cytoplasmic effectors, primarily produced by bacteria, influence plant responses and disease symptoms (Todd et al., 2022). They achieve this by interfering with gene transcription and targeting susceptible factors, which facilitates pathogen growth. One such group of cytoplasmic effectors is the transcription activator-like effectors (TAL) from *Xanthomonas*, which alter plant transcription factors. TAL effectors are secreted by the type III secretion system. The RxLR effector, produced by *Phytophthora*, exhibits pathogenicity and suppresses host defense (Jiang et al., 2008). Some effectors hijack the host cell machinery by mimicking host cell proteins. Phytoplasmas produce effector molecules, such as SAP (secreted aster yellows witches' broom proteins), which target host transcription factors like TCPs (teosinte branched/cycloidea/proliferating cell factor) and RAD23, thereby altering host development and immunity (Janik et al., 2017).

Apoplastic effectors, which are produced by fungi, insects, and nematodes, are characterized by their secretory nature. One such effector is Ecp20-2 produced by *Cladosporium fulvum*, (Stergiopoulos et al., 2010; Westerink et al., 2004; Van Esse et al., 2007) which inhibits the production of plant enzymes, detoxifies reactive oxygen species, and suppresses PAMP-triggered immunity (Chen et al., 2023). Table 1 provides a list of effector molecules that can be identified and targeted for innovative and improved disease management strategies.

**Table 1:** Effectors produced by different plant pathogens during pathogenesis.

**Effectors**	**Plant pathogens**	**Reference**
**Bacteria**		
TAL	*Xanthomonas oryzae* pv. *oryzae, Xanthomonas axonopodis* pv. *citri, Xanthomonas axonopodis* pv. *glycines*	Zhang et al., 2015
	*Xanthomonas translucens* pv. *undulosa*	Peng et al., 2019
	*Xanthomonas axonopodis pv. manihotis*	Wang et al., 2024
PthA	*Xanthomonas axonopodis* pv. *citri*	Swarup et al., 1991
Avrb6, PthN	*Xanthomonas axonopodis* pv. *malvacearum*	Yang et al., 1996; Chakrabarty et al., 1997
AvrBs1, AvrBs2, avrBs3	*Xanthomonas axonopodis* pv. *vesicatoria*	Kearney et al., 1988, Marois et al., 2002, O'Garro et al., 1997
AvrXa5, AvrXa7, PthXo3	*Xanthomonas oryzae* pv. *oryzae*	Li et al., 2018a,b
AvrPtoB	*Pseudomonas syringae*	Shan et al., 2000
PsyB728a, HopA1	*Pseudomonas syringae* pv. *syringae*	Kang et al., 2021
AvrRpm1	*Pseudomonas syringae* pv. *maculicola*	Ritter and Dangl, 1995
AvrPphF, virPphA, AvrPphC	*Pseudomonas syringae* pv. *phaseolicola*	Tsiamis et al., 2000, Yucel et al., 1994
AvrA, AvrE, AvrPto, AvrRpt2	*Pseudomonas syringae* pv. *tomato*	Shan et al., 2000
Rip36, RipAB, Rip1, RipAY, RipAX2, RipB, RipJ, RipAZ1, RipAL	*Ralstonia solanacearum*	Nakano and Mukaihara, 2019; Pandey et al., 2021; Moon et al., 2021; Nakano and Mukaihara, 2018
dspEF	*Erwinia amylovora*	Bogdanove et al., 1998
SAP11 SWP1, SWP12, SWP21, SWP11	Aster Yellows phytoplasma	Lu et al., 2015; Wang et al., 2018
SAP54	*Candidatus* Phytoplasma australasia, Bellis virescence phytoplasma	Ahmed et al., 2022; MacLean et al., 2011
**Fungi**		
AvrM	*Melamspora lini*	Ve et al., 2013
AvrPia, AvrPik, PWT3	*Magnaporthe oryzae*	Cesari et al., 2013, Kanzaki et al., 2012, Inoue et al., 2017
Avramr3, Pi02860, Pi04314/RD2, Pi04314/RD24, PiAvr2	*Phytophthora infestans*	Lin et al., 2022a,b; He et al., 2018; Yang et al., 2016; Boevink et al., 2016; Gilroy et al., 2011; Turnbull et al., 2019
PsAvh52	*Phytophthora sojae*	Li et al., 2018a,b
AlAvr1	*Ascochyta lentis*	Henares et al., 2022
AvrRppC	*Puccinia polysora*	Deng et al., 2022
SsITL	*Sclerotinia sclerotiorum*	Zhu et al., 2013; Tang et al., 2020
Pst18363, PstGSRE4, PstGSRE1, Pst_12806	*Puccinia striiformis* f. sp. *tritici*	Xu et al., 2019; Qi et al., 2019; Liu et al., 2022
SCRE6	*Ustilaginoidea virens*	Zheng et al., 2022
Umrip1	*Ustilago maydis*	Chan, 2022
ToxA, PtrToxB	*Pyrenophoratritici repens*	Friesen and Faris, 2004; Figueroa et al., 2015
SnTox1	*Parastagnospora nodorum*	Liu et al., 2012

S gene, Susceptibility gene; TAL, Transcription Activator Like; Pth, Pathogenicity; Avr, Avirulence; Rip, Ralstonia Injected Proteins; dsp, Disease Specific Protein; SAP, Secreted Aster Yellows Witches' Broom Protein.

### 2.2 Improving plant disease management through effector-directed interventions

Resistance achieved through conventional breeding methods can be overcome by pathogens, which generate new, more virulent strains (Shang et al., 2023). In contrast, strategies for targeting effectors for plant disease management offer several promising advantages. First, these strategies can alter pathogenicity and affect the virulence of the pathogen to some extent (Todd et al., 2022). Second, most of the effectors are conserved among multiple pathogenic strains, making them an ideal target for broad-spectrum activity (Sha and Li, 2023). For instance, Avr (avirulence) and RxLR effectors are conserved across various pathogens offering durable resistance to varied pathogens in most crops. Third, targeting site-specific effectors could reduce off-target effects on beneficial microbes in ecosystems. Finally, strategies for effector targeting are compatible with other disease management methods, which could lead to a sustainable, multi-pronged approach in the future.

### 2.3 Methods of targeting effector proteins

**Figure 1:**
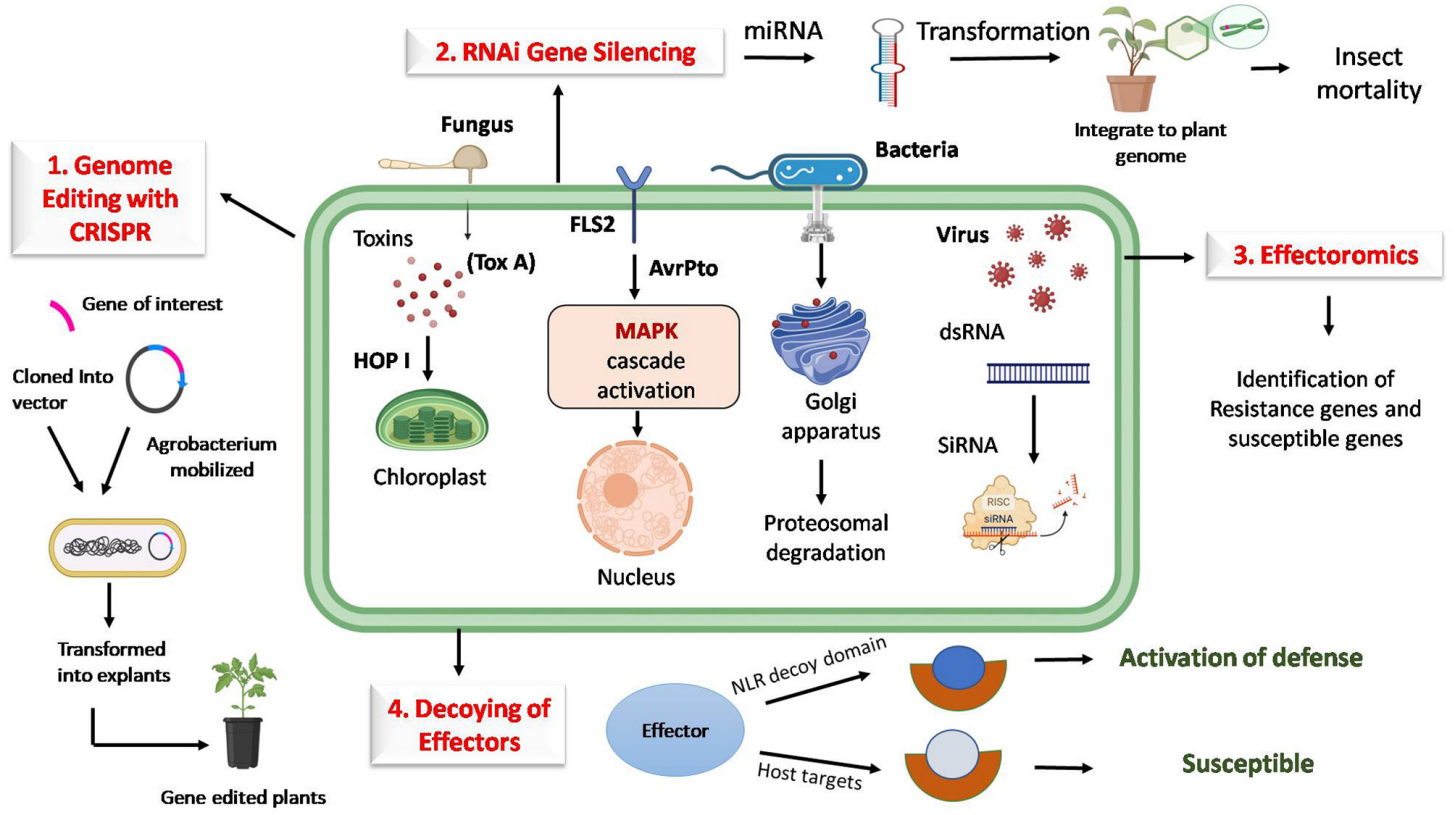
Strategies for targeting host factors to manipulate pathogen effectors for durable plant disease resistance. (1) Genome editing with CRISPR/Cas targets plant genes by impairing effector targets, which disrupts pathogen compatibility. (2) RNA interference (RNAi) enables host-induced gene silencing of effector genes. (3) Engineering effector decoys mimics effector targets and intercepts pathogen effectors. (4) Effectoromics-based identification of gene pathways and networks manipulated by effectors, providing precise intervention points, thereby providing resistance. Together, these approaches offer a layered defense strategy that interrupts the pathogen's effectors and provides durable resistance to host plants.

Effectors can be targeted using various biotechnological approaches, such as genome editing tools, RNA interference, effector decoy strategies, and effector breeding and diagnostics. Various -omics approaches can be used to understand the molecular level of these effectors and improve precision management, as shown in Figure 1.

#### 2.3.1 Genome editing with CRISPR/Cas

Genome editing offers two complementary approaches: disruption of effector binding elements (EBEs) in the promoter regions of host susceptible genes and knocking out negative regulators. By modifying EBEs through mutation, for instance, we can prevent effector binding and subsequent activation of the target site, thereby inhibiting pathogenicity, virulence, recognition, and colonization by the pathogen. For example, the SWEET (sugar will eventually be exported transporter) genes are known susceptibility genes (S genes) to which TAL effectors bind at specific EBEs in the promoters of these genes, leading to their overexpression. Sugar efflux into the apoplast provides the pathogen with nutrients, thereby enhancing infection and disease progression. CRISPR/Cas can be used to edit SWEET genes (OsSWEET11, 13, and 14) to disrupt EBEs in their promoters confers resistance against bacterial leaf blight in rice (Zhou et al., 2015). In cassava, the SWEET10a gene targets host genes that increase the resistance toward *Xanthomonas axonopodis* pv. *manihotis* (Wang et al., 2024). This prevents TAL effector-mediated activation and confers resistance to bacterial blight in elite rice cultivars (IR64, Ciherang-Sub1, and Kitaake). Disrupting TAL-EBEs blocks the pathogen-induced gene activation and enhances blight resistance without affecting plant development (Li et al., 2025). Second, the Mildew Locus O (MLO) gene family encodes membrane-associated proteins that negatively regulate plant defense responses. These genes are well-characterized susceptibility genes in both monocots and dicots, as loss-of-function mutations in MLO result in broad-spectrum resistance to powdery mildew pathogens. Using CRISPR/Cas9, targeted knockouts or frameshift mutations in MLO genes have been achieved in species such as wheat, tomato, and grapevine. This reduces or eliminates functional MLO protein activity and thereby confers resistance without significant developmental penalties (Nekrasov, 2019). In both banana and tomato plants, knocking out the DMR6 gene led to increased resistance to *Xanthomonas* (Tripathi et al., 2021; Thomazella et al., 2021). Similarly, the transgenic expression of the Bs2 gene from pepper detects the effectors produced by *Xanthomonas*, thereby providing resistance.

Although targeted genome editing can provide durable resistance, identifying S genes is challenging because they are often recessive and have multiple copies, unlike resistance genes. Identification methods are thus time-consuming and labor-intensive, often relying on wild cultivars to achieve optimal results. Furthermore, targeting S genes is known to have pleiotropic effects, including negative effects on plant growth and yield. This is undesirable for disease management in agriculture. Validating these effectors as S genes highlights the need to balance pathogen specificity with agronomic performance.

#### 2.3.2 RNA interference and gene silencing

RNAi-mediated silencing enables the direct targeting of pathogen effector molecules either through host-induced gene silencing (HIGS) or spray-induced gene silencing (SIGS). HIGS is durable and can silence multiple effectors simultaneously, but it relies on stable transgenics, which pose regulatory challenges. On the contrary, SIGS provides a non-transgenic and eco-friendly alternative, but it depends on the stability and delivery efficiency of dsRNA. Compared to CRISPR, RNAi offers greater flexibility in targeting multiple effectors, but it lacks the long-term durability of genetic modifications, making RNAi a suitable option as an interim strategy. There are reports that RNAi is successful in silencing the effector genes of plant-parasitic nematodes, such as *Meloidogyne incognita*, leading to reduced infectivity (Shivakumara et al., 2016). In *M. incognita*, RNAi targets and suppresses genes such as msp-18, msp-20, msp-24, msp-33, and msp-16. These genes interact with host transcription factors by altering the expression of cell wall-degrading enzymes (Shivakumara et al., 2016). Putative effectors in the nematode, *Pratylenchus thornei* were identified, and upon introducing RNAi, they exhibited severe effects on phenotype, behavior, gene expression, and the reproductive system (Khot, 2018). Similar effects were observed using RNAi in the fungal pathogens, such as *Fusarium, Verticilium*, and *Rhizoctonia* (Foroud et al., 2014), as well as in insect vectors, including whiteflies and aphids (Feng et al., 2023). Host plants adopt a mechanism of host-induced gene silencing when they use RNAi molecules. This mechanism targets and silences specific effectors, thereby reducing the pathogen's virulence and inhibiting colonization. This reduces pest and disease incidence and provides better management strategies.

#### 2.3.3 Decoying of effectors

Decoy engineering converts susceptible nature into resistance by providing plants with engineered proteins that mimic natural effector targets, sequestering effectors before they interact with host proteins. When the pathogens bind to the decoys, they are prevented from reaching their actual targets within the host, thereby suppressing pathogen infection. This approach is highly specific once the effector-target interaction is well-established. These decoys prevent the effectors from reaching their EBEs, a mechanism that has been well-documented in R genes, which provide host plants with broad-spectrum resistance. In the future, synthesizing such decoys could provide an opportunity to design novel resistance strategies based on specific EBEs.

#### 2.3.4 Effectoromics

Effectoromics is a potentially powerful approach for quickly and efficiently identifying novel R genes. Pathogen effectors act as tools that identify resistance genes across germplasm collections through immune response screening (Domazakis et al., 2017). They also differentiate functional redundancy and specificity. These R genes form the basis for breeding methods that increase resistance and incorporate effector-triggered immunity into crop improvement programs. Similarly, R genes such as Rpi-amr4, Rpi-amr16, and Rpi-amr17 were identified in potatoes in response to the late blight pathogen, *Phytophthora infestans* effector RxLR genes Avramr4, Avramr16, and Avramr17 (Lin et al., 2022a,b). These genes act as resistance genes in the host plant and are used for effective disease management. However, this approach is data-intensive and functional validation of candidate susceptible genes remains time-consuming; it does not confer resistance, but serves as an indispensable backbone that informs and strengthens effector targeting strategies.

## 3 Conclusion

The major current and future challenges in agriculture on a global level are emerging plant diseases, pathogen resistance, and climate change. Hence an urgent need for innovative, cost-effective and sustainable solutions is critical. Targeting effectors is durable and eco-friendly, disabling the limitations of chemical-based management, such as emerging pathogen resistance and harm to the beneficial microbiome within the ecosystem. Targeting effectors disarms the pathogen at the molecular level, modifying the strategy toward an ecologically based approach to crop protection. Leveraging new technologies such as genome editing, RNA interference (RNAi), decoying of effectors, and effectoromics can advance plant disease management results, which face uncertainties in durability, delivery efficiency and environmental stability. An effector-based approach could be the future technology, transforming plant pathology into a science driven by prediction and precision rather than reaction. However, biosafety and ecological considerations such as unintended impacts on beneficial microbes or non-target organisms must be critically evaluated. This shift would help to secure global food security by enabling the development of disease resistant varieties. Further, to translate these approaches into practical crop improvement, it requires integration of effectoromics into breeding pipeline, their validation under field conditions, incorporating with integrated plant disease management provides a path forward, ensuring that effector targeting strategies can make a meaningful contribution to global food security.
